# Effects of hypoxia and nanocarrier size on pH-responsive nano-delivery system to solid tumors

**DOI:** 10.1038/s41598-021-98638-w

**Published:** 2021-09-29

**Authors:** M. Soltani, Mohammad Souri, Farshad Moradi Kashkooli

**Affiliations:** 1 Department of Mechanical Engineering, K. N. Toosi University of Technology, Tehran, Iran; 2 Department of Electrical and Computer Engineering, University of Waterloo, 200 University Ave., Waterloo, ON N2L3G1 Canada; 3 Centre for Biotechnology and Bioengineering (CBB), University of Waterloo, Waterloo, ON Canada; 4 Advanced Bioengineering Initiative Center, Computational Medicine Center, K. N. Toosi University of Technology, Tehran, Iran

**Keywords:** Drug delivery, Nanotechnology in cancer, Targeted therapies, Targeted therapies, Cancer microenvironment, Computational models, Cancer models

## Abstract

One of the special features of solid tumors is the acidity of the tumor microenvironment, which is mainly due to the presence of hypoxic regions. Therefore, pH-responsive drug delivery systems have recently been highly welcomed. In the present study, a comprehensive mathematical model is presented based on extravascular drug release paradigm. Accordingly, drug delivery system using pH-responsive nanocarriers is taken into account to examine the impacts of hypoxic regions as well as the size of nanocarriers for cancerous cell-death. The extent of hypoxic regions is controlled by vascular density. This means that regions with very low vascular density represent regions of hypoxia. Using this mathematical model, it is possible to simulate the extracellular and intracellular concentrations of drug by considering the association/disassociation of the free drug to the cell-surface receptors and cellular uptake. Results show that nanocarriers with smaller sizes are more effective due to higher accumulation in the tumor tissue interstitium. The small size of the nanocarriers also allows them to penetrate deeper, so they can expose a larger portion of the tumor to the drug. Additionally, the presence of hypoxic regions in tumor reduces the fraction of killed cancer cells due to reduced penetration depth. The proposed model can be considered for optimizing and developing pH-sensitive delivery systems to reduce both cost and time of the process.

## Introduction

Hypoxia is one of the most important features of solid tumors that affect tumor growth as well as therapeutic responses^1,2^. Most of the solid tumors have a similar metabolic process, since they first use the blood supply system of the host tissue to provide their oxygen and nutrients^3^. As the tumor grows, the need for oxygen and nutrients increases to meet the demands of the tumor. Hence, tumors form their own microvascular network from the host circulatory system through angiogenesis^4,5^. However, the formed microvasculature is chaotic and suffers from functional and structural abnormalities^6,7^. Thus, this microvascular network forms oxygen deficiency areas (i.e., hypoxic regions) inside the solid tumor, especially at central parts of the tumor^8,9^. It is reported that more than 50% of solid tumor cells, mostly those that are located in the tumor central areas, are deprived of sufficient oxygen supply; while, the cells that are placed close to tumor periphery are adequately oxygenated^10–12^. Hypoxic regions are found at different stages of tumor growth, for example in some cases in malignant tumors that are only 1 mm in size^13^. As cells need oxygen and adequate amounts of nutrients for metabolic activities, oxygen deficiency is a major challenge for cell survival^14,15^. However, cancer cells intelligently adapt to the hypoxic stress through a sequence of hypoxia-induced factors (HIF), mainly HIF-1, and undergo genetic transformations^16^. This is a disaster, because it helps the disease to progress via reconstruction the extracellular matrix (ECM)^17^.

Another important feature of solid tumors, which is traditionally associated with hypoxia, is the acidity of the tumor microenvironment (TME), i.e., lack of oxygen and acidity have been observed simultaneously in the TME^18^. Acidic TME is arising from hypoxia regions and accumulation of extracellular lactic acid in tumors^19,20^. In addition, pumping protons from hypoxic tumor cells further lowers the surrounding pH. Other metabolic processes, including tumor respiration, transport of ${H}^{+}$ and $HC{O}_{3}^{-}$, and extracellular drainage of ${H}^{+}$ also affects tumor acidity^19,20^. In general, high glucose metabolism rate in cancer cells under aerobic and anaerobic conditions causes acidification of tumor tissue compared to normal tissue^21^. Therefore, the pH gradient is a special feature of tumor that is of great importance in using targeted pH-responsive nano-sized drug delivery systems for tumor treatment^22^. Nano-based drug delivery systems encapsulate anti-cancer drugs in the carrier, which significantly reduces the side effects of anti-cancer drugs compared to their free injection. Also, due to surface-area-to-volume ratio of nanocarriers, they have a high loading capacity^9^. On the other hand, by engineering the physical and chemical properties of nanocarriers, their non-specific distribution can be prevented. Among these, the size of nanocarriers is one of the most important physicochemical factors that determines the amount of accumulation in the tissue. Nanocarriers are mainly designed in sizes over 12 nm, as the nanocarriers should be larger than the gap between the endothelial cells of the normal tissue microvasculature (less than 6 nm) to prevent accumulation of nanoparticles in normal tissue^22,23^. Therefore, it is necessary to evaluate this factor for different purposes to achieve the best performance of nano drug delivery system.

For effective delivery of encapsulated anticancer drugs via pH-responsive carriers, it is necessary to store and stabilize the drug-loaded pH-sensitive nanocarrier at physiological pH level; while, drug will be released at target site by reaching the pH level of carrier to trigger point^24^. According to the composition of nanocarriers, pH-responsive nanocarriers can be classified into organic, inorganic, and hybrid^25^. Drug release strategies vary in response to pH based on the composition and structure of the nanocarriers; however, among these factors, the swelling and solubility release mechanisms play a prominent role^9^, which fall into the category of sustainable release because drugs are released over a long period of time due to very low release rates^9^. Sustainable release mechanism increases the effectiveness of drugs that are rapidly metabolized and excreted, because by stabilizing the concentration level, the cancer cell is exposed to an appropriate drug concentration for a long time, preventing tumor growth through controlling cell proliferation^9,26^.

**Figure 1:**
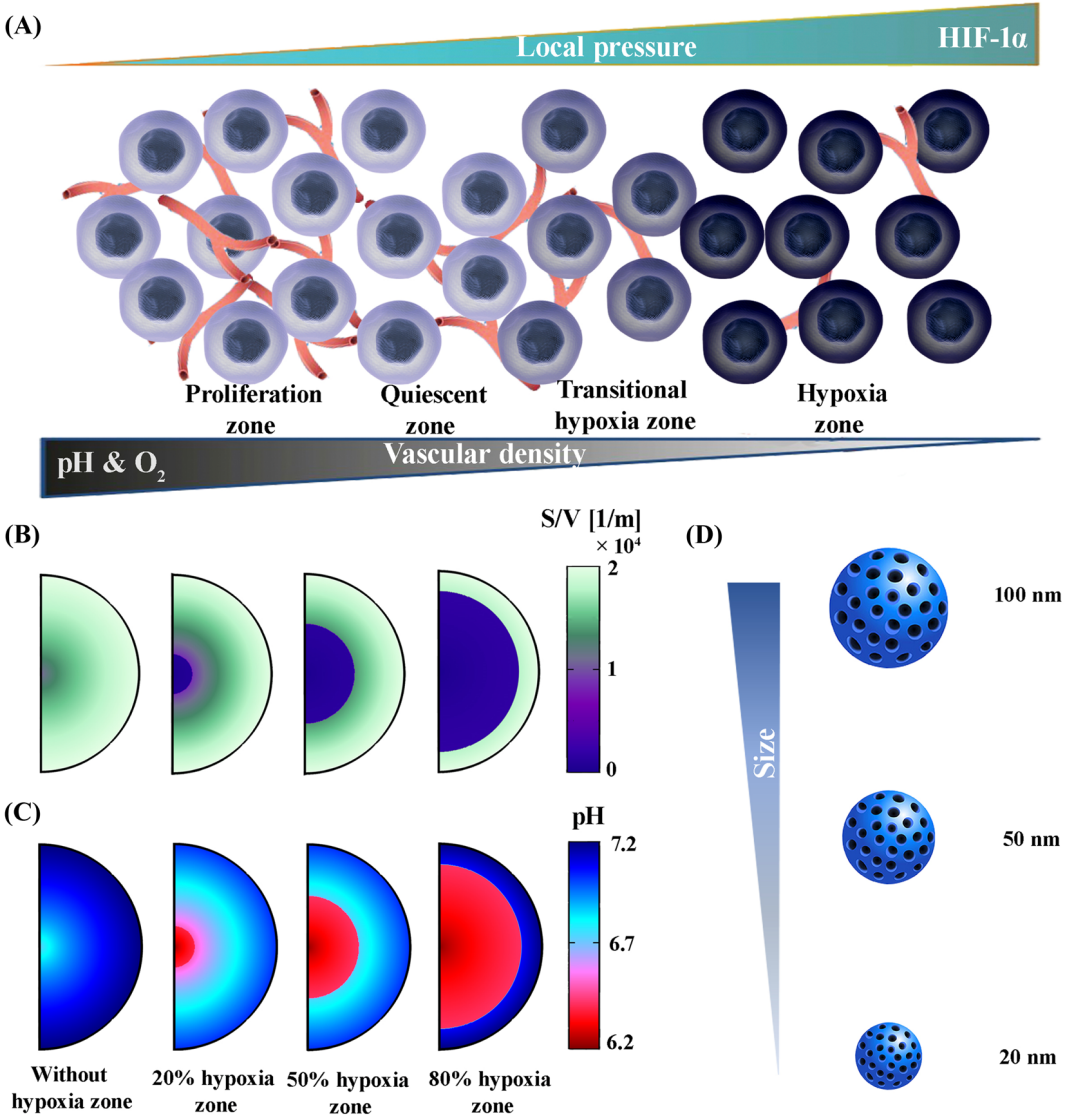
An overview of the issues examined in this study; (**A**)The proliferative region, which contains the highest vascular density, has a higher oxygen level and consequently, a lower acidity level, unlike the hypoxic region, which has the lowest vascular density. Quiescent and proliferation regions do not suffer from lack of oxygen. Also, the only difference between these two regions is related to their vascular density so that the proliferation regions has a higher vascular density, (**B**) Based on the hypoxic region, which has the lowest vascular density, 4 different cases are considered for solid tumors (Without hypoxia region, 20% hypoxia zone, 50% hypoxia zone, and 80% hypoxia zone), (**C**) Hypoxic region has the lowest pH (6.2) and the tumor periphery has the highest pH (7.2). Acidity levels in other regions of the tumor depending on vascular density, (**D**) pH-responsive nanocarriers in various sizes of 20, 50, and 100 nm are taken into account to analyze the effect of nanocarrier size.

In the present study, for the first time, a pH-responsive nano-sized delivery system based on the extravascular release paradigm is investigated through a developed mathematical model. Figure 1 presents an overview of the issues examined in this study. The negative effects of the hypoxic region on the therapeutic response have been proven due to their very poor perfusion in solid tumors. Based on vascular density, which represents perfusion, solid tumors are divided into three different zones, including proliferation $(S/V \ge 150[1/M])$, quiescent $(S/V \ge 100[1/M])$, and hypoxia $(S/V \ge 20[1/M])$. The vascular density of the hypoxic regions is less than 0.1 of that in the proliferation zone. Areas with a vascular density of $20[1/M] \le S/V \le 100[1/M]$ are known as transitional hypoxic regions. Here, $S$ is the vascular surface area and $V$ is the volume of the tissue, which $S/V$ is referred to as vascular density. In the proliferative regions, angiogenesis process is still ongoing and vascular density is also increasing; while, in the quiescent regions, angiogenesis is perfectly finished. Based on vascular density and hypoxic regions, 4 different cases are evaluated for solid tumor (Fig. 1B). On the other hand, due to the density distribution of blood microvessels, which also shows the oxygen level, the pH level of the tumor varies. According to the literature, the hypoxic region has the lowest pH (5.7–6.5)^27,28^. However, in the proliferative region, due to the high density of blood microvessels, H^+^ ions are washed out by venting into the bloodstream, which raises the pH compared to the other areas (7–7.4) (Fig. 1C)^20^. Therefore, the pH gradient range of tumor extracellular varies between 6.2 and 7.2. So, the pH-responsive nanocarriers have different release rates, depending on the location. Hence, in this study, the effect of nanocarrier size as an important physicochemical property on biological distribution is investigated. Also, assuming equal amount drug-loading, the therapeutic results are investigated (Fig. 1D). The size of the nanocarriers has been selected by considering hydrodynamic and electrostatic interactions as the nanocarriers pass through vascular pores of a specified size (200 nm) in the appropriate range (≤ 100 nm)^22,29,30^.

## Results and discussion

### Fluid flow

**Figure 2:**
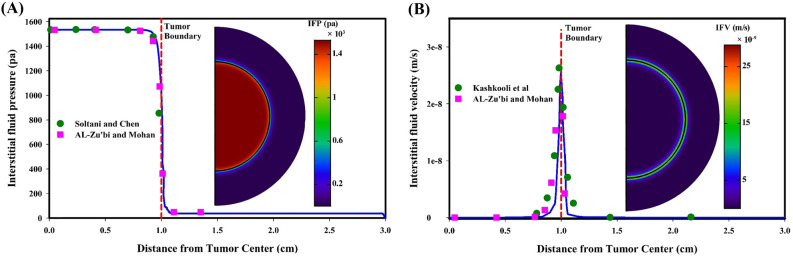
Interstitial fluid fields; (**A**) IFP is quickly dropped at the tumor boundary, while has uniform distribution in other areas. IFP prediction in the present model has been compared with Soltani and Chen^31^ and Al-Zu’bi and Mohan^33^ models, showing high compatibility of the results; (**B**) IFV is rapidly increasing at the tumor boundary, while it is minimal in other areas. IFV prediction in the present model has been compared with the models of Kashkooli et al.^36^ and Al-Zu’bi and Mohan^33^. The discrepancy between the current model and other models can be related to the vascular density and vascular distribution.

Interstitial fluid fields play a significant role in the transport of therapeutic agents in biological tissues, because both interstitial fluid pressure (IFP) and interstitial fluid velocity (IFV) determine the migration of therapeutic agents in the ECM through convection mechanism. Additionally, the difference between IFP and intravascular pressure in the microvascular network is an influential factor in the transvascular exchange of therapeutic agents. According to the properties of biological tissues, Darcy law is employed to simulate the flow behavior^31^. Accordingly, for the condition of 2080 Pa in intravascular pressure^32^, the distribution of IFP and IFV in both tumor and healthy tissues is demonstrated in Fig. 2. IFP is uniform in both tumor and normal tissues, whereas there is a large pressure gradient in a narrow layer between tumor and healthy tissue. It is also clear that the tumor IFP is much higher than that of normal tissue. The reason is that due to the dysfunctional lymphatic system in the tumor, the microvascular network of the tumor has a higher leakage rate than that of normal tissue, i.e., the material entering the interstitial space do not leave it, so they accumulate in the tumor tissue and increases the IFP. The mean spatial value of tumor IFP is obtained 1533.88 Pa; which, in addition to agreeing with previous numerical studies^31,33^, also has good compatibility with experimental study^34^ that show the tumor IFP is placed in the range of 586 to 4200 Pa. The spatial mean value of IFP in normal tissue is 40 Pa; which, in addition to numerical studies, agrees with experimental test^35^ in which an IFP in normal tissue has the range of -400 to 800 Pa. In contrast to IFP, the mean value of IFV for both tumor and normal tissues has the lowest value. Maximum IFV occurs only at tumor margins where there is a large IFP gradient. According to Fig. 2B, the IFV is an order of ${10}^{-8}$ m/s that has not been recorded by experimental studies, so it has been compared with the previous numerical models, demonstrating a good agreement between the results.

### Therapeutic agents transport and therapeutic response

Drug-loaded pH-responsive nanocarriers do not release the drugs in the bloodstream and normal tissue due to their neutral pH level. After extravasation of the nanocarriers from the vessels into the tumor interstitium, they react in response to the acidic TME and release the drug at a certain rate based on carrier site, i.e., pH distribution in tumor. On other hand, vascular density determines the spatial accumulation of nanocarriers in TME. Transport of therapeutic agents in the interstitium is determined through the convection–diffusion-reaction (CDR) equations. Responsive nanocarriers and drug molecules penetrate deep into the tumor based on the concentration gradient, known also as the inward diffusion mechanism. However, the outward interstitial fluid flow resists against depth of penetration based on the convection mechanism, although its resistance level is not strong. However, the size of nanocarriers is a determinant factor in efficacy of nano-size drug delivery system, which has great impact on the penetration depth. This is due to the size ratio of nanocarriers to ECM pores, they have a very poor diffusion coefficient in interstitium. In the following nano-based drug delivery process, released free drugs bind to cell-surface receptors with a high binding affinity. If they are not unbound by the receptors, they are internalized to the cells and cause cell-death by damaging the organelles of the cell.

**Figure 3:**
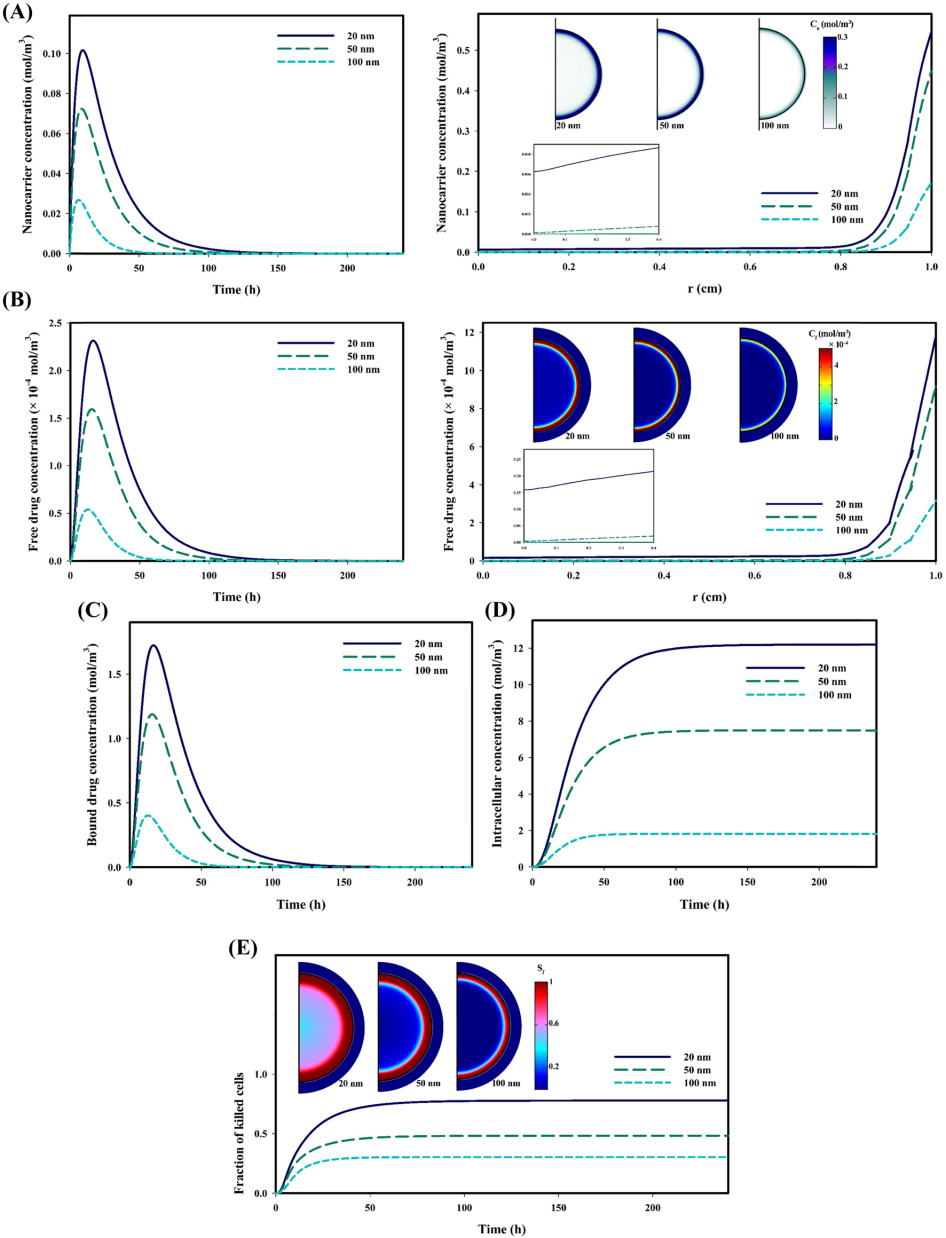
Distribution of therapeutic agents and therapeutic response in a vascularized tumor for three different sizes of pH-responsive nanocarriers; (**A**) Spatiotemporal distribution distribution of responsive nanocarriers in tumor interstitium; Smaller nanocarriers have more accumulation in tumor tissue and also have a higher penetration depth, (**B**) Spatiotemporal distribution distribution of free drug in tumor interstitium; The concentration of released free drugs from smaller nanocarriers is greater due to the high accumulation of smaller nanocarriers compared to their larger counterparts, (**C**) Temporal distribution of bound drug in tumor interstitium; High accumulation of drugs released from 20 nm nanocarriers increases the chance of free drug molecules binding to cell surface receptors, (**D**) Temporal distribution of internalized drug in cellular; Binding of high concentrations of free drug molecules to cell surface receptors results in higher concentrations of internalized drug, (**E**) Fraction of killed tumor cells over time; High concentration of internalized drugs increase the fraction of killed cells. Also, due to the accumulation of free drug in the periphery of the tumor, the cells in this region have suffered the most damage. (${C}_{n}$: Nanocarrier concentrations ,${C}_{f}$: Free drug concentrations , ${S}_{f}$: Fraction of killed cells).

Figure 3 shows the distribution of therapeutic agents as well as therapeutic response when the tumor is not affected by hypoxia regions, i.e., in this case, all areas of the tumor have good perfusion. Figure 3A shows the temporal distribution of nanocarriers in different sizes and their spatial distribution when the maximum concentration is recorded. Using nanocarriers in a size of 20 nm, due to higher permeability, accumulate more in the tumor interstitium; while, employing 100 nm nanocarriers lead to the lowest accumulation. Additionally, carriers with a size of 20 nm have a higher penetration depth that allows them able to reach the tumor center, although their concentration is not significant in the tumor center. In contrast, 100 nm nanocarriers have a weak accumulation in a narrow layer at the tumor periphery. Carriers begin to release the drug in response to the acidic environment; however, due to the very low release rate, the maximum concentration of released drug is recorded about 10 h after the maximum concentration nanocarriers is happened. Figure 3B shows the temporal distribution of the free drug in different sizes and their spatial distribution when the maximum concentration is recorded. As expected, due to assuming equal amount drug-loading and higher accumulation of nanocarriers with smaller sizes because of longer half-life of smaller nanocarriers, the accumulation of free drugs delivered by 20 nm carriers is higher. Despite these interpretations, the penetration depth of the free drug is weak due to the high binding affinity; i.e., the rapid binding of the released drug molecules to the cell-surface receptors in each area of the tumor causes the drug penetrate less deeply into the tumor. The temporal distribution of the bound drug has similar trend with that of the free drug, and the maximum concentration is occurred when 20 nm nanocarriers are used to deliver the drug (Fig. 3C). Low rate of the transmembrane prolongs the internalization process of the drug. Figure 3D shows the temporal distribution of the internalized drug in different sizes. As expected, the concentration of the internalized drug has the highest value when the anticancer drug is delivered by 20 nm nanocarriers; while, the delivery by 100 nm nanocarriers has the lowest intracellular concentration (Fig. 3D). Once the drug molecules are internalized to the cellular space, they damage cell organs and cause cell-death, due to their high metabolism as well as ignoring the effects of multidrug resistance (MDR). Figure 3E shows the fraction of killed cells for different sizes of nanocarriers. It is clear that a high concentration of the intracellular drug, in the case of using 20 nm carriers, increases the fraction of killed cells by about 0.77. Additionally, accumulation of free drugs in the tumor periphery and the proliferative region also causes the cells of this region to be more susceptible to death.

**Figure 4:**
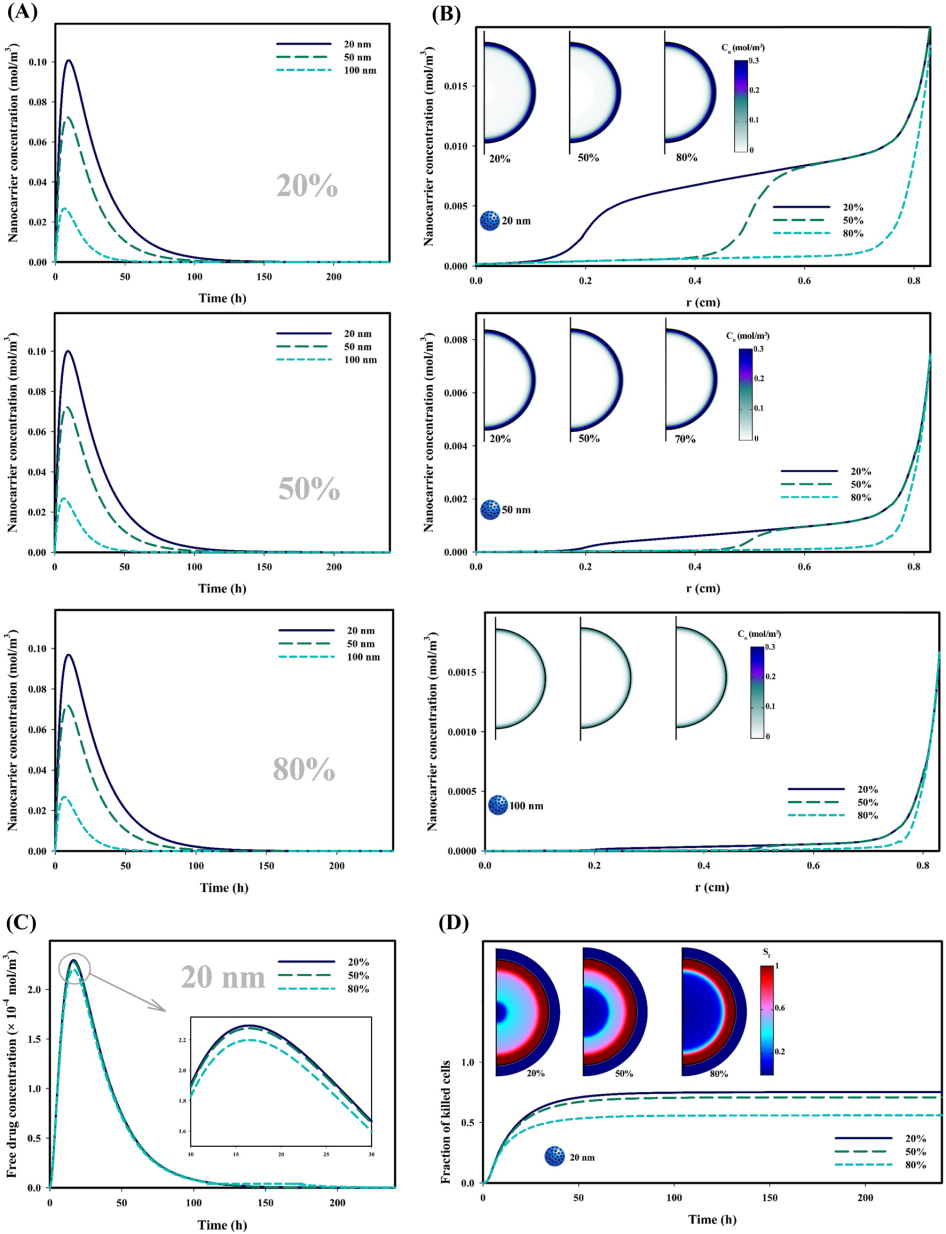
Influence of hypoxic region on the distribution of therapeutic agents and therapeutic response; (**A**) Temporal distribution of responsive nanocarriers in tumor interstitium; Concentration of smaller nanocarriers is higher in tumor tissue, and the increase in the extent of the hypoxic region reduces the concentration, especially for small nanocarriers, (**B**) Spatial distribution of responsive nanocarriers in tumor interstitium; The highest concentration of nanocarriers is in the periphery of the tumor, while the hypoxic regions have the lowest concentration due to poor perfusion, (**C**) Temporal distribution of drug released from nanocarriers (20 nm) in tumor interstitium; Weaker accumulation of nanocarriers in tumors involving larger hypoxic regions, results in lower concentrations of free drug, (**D**) Fraction of killed tumor cells over time; Due to the fact that the concentration of the free drug in hypoxia areas is very low, so the cells in these areas do not suffer from death due to anti-cancer drug. (${C}_{n}$: Nanocarrier concentration, ${S}_{f}$: fraction of killed cells).

Anticancer drugs reach their highest therapeutic potential through enough accumulation in the interstitium for a long time and reaching the tumor center to engage the whole tumor. As discussed, the spatial distribution of vascular density in tumor determines the extent of local accumulation. Among all types of tumors at any stage of their growth, tumors affected by hypoxia suffer from poor vascular density. Figure 4 investigates the effect of hypoxic regions on the therapeutic agents’ distribution as well as therapeutic response. Figure 4A shows the temporal distribution of nanocarriers in different sizes for three various tumor states affected, respectively 20%, 50%, and 80% by hypoxia. Results demonstrate that concentration level of the carriers has changed very little in all three cases, except for 20 nm in which the concentration level changes very slightly. This phenomenon is interpretable according to Fig. 3A, as nanocarriers are mainly concentrated in the proliferative region, where there are no hypoxic regions. It should also be noted that the distribution of 100 nm carriers is not influenced by 80% hypoxic regions in the tumor, because they have very poor penetration depth. Figure 4B shows the spatial distribution of carriers, in which concentrations of injected carriers are generally low in central areas of the tumor, where hypoxia is considered; however, the concentrations for 50 and 100 nm-sized carriers are much less than 20 nm. An important point extracted from Fig. 4B is the sharp decrease in the concentration of carriers with sizes of 20 and 50 nm in hypoxic regions due to their poor vascular density. However, this phenomenon is not observed in 100 nm carriers due to poor penetration.

Given the phenomena that have taken place, it is expected that hypoxic regions have a significant effect only on the biodistribution of carriers with a size of 20 nm. Therefore, Fig. 4C shows the temporal distribution of free drug, which depends on the distribution of the carrier. It is known that decrease in vascular density as well as perfusion caused by hypoxia regions reduces the level of free drug concentration, especially in the case that 80% of the tumor has been affected by the hypoxia. As it turned out, hypoxic regions reduce the depth of penetration; so, less tumor volume is exposed to the drug, leading to a reduction in the fraction of killed cancerous cells, as shown in Fig. 4D. It is clear that in hypoxic regions where the drug concentration is much lower, the fraction of killed cells parameter has also the lowest value. Thus, when 80% of the tumor has been affected by the hypoxia, more than 25% of the tumor cells survive compared to the vascularized tumor.

Another important point that can be understood from Figs. 3 and 4 is the explanation of effects of therapeutic agents on the host tissue of tumor, which also shows the side effects of drug delivery system. Due to the high ratio of the size of the nanocarriers to the vessel-wall pores of normal tissue, permeability value is zero. Hence, the free drug released from responsive nanoparticles is not found in the interstitium of normal tissue, just found in a narrow layer at the border between normal and tumor tissues. Indeed, therapeutic agents that are pushed out from the tumor to the periphery area due to the convection mechanism are found in normal tissue, which cannot significantly damage normal tissue.

## Conclusions

In this study, using a mathematical model and considering the biological characteristics of tumor tissue ─such as the distribution of acidity and hypoxia regions ─, the transport of drug-loaded pH-responsive nanocarriers are investigated for the first time. By simulating the extracellular and intracellular concentrations of the anticancer drug and considering the association/disassociation of the free drug to the cell-surface receptors as well as cellular uptake, drug delivery efficacy is determined through the fraction of killed cells parameter. In this study, three pH-responsive nanocarriers ─with sizes of 20, 50, and 100 nm─ have been used to examine their impacts on drug delivery. Additionally, the effect of hypoxic regions ─with sizes of 20%, 50%, and 80% of tumor size─ on drug delivery is investigated considering different size of carriers. It is found that carriers with a smaller size (20 nm), due to high permeability, are able to accumulate higher in the tumor tissue; therefore, the extracellular concentration of free and intracellular drugs is high. Thus, it can cause the death of more than 75% of cancer cells. However, only 30% of cancer cells are killed in delivery of the drug by 100 nm nanocarriers, due to poor accumulation of the free drug which is exchanged between the extracellular and intracellular spaces. Additionally, the study of the effect of hypoxic regions shows that poor perfusion, due to poor vascular density, reduces the accumulation of carriers in the tumor interstitium. Hence, if 80% of the tumor affected by hypoxia, 25% less cell-death occurs compared to the vascularized tumor. This is true for 20 nm nanocarriers, because they have a greater penetration depth compared to other investigated sizes. The proposed in silico study can help optimize and develop pH-sensitive drug delivery systems by considering the characteristics of TME. Therefore, by reducing clinical trials and the number of animals used in biomedical studies, researchers can save both time and cost.

## Method

### Mathematical model

The mathematical model of drug delivery consists of:

- (*i*) Mass and momentum conservations for interstitial fluid flow to obtain IFP and IFV;
- (*ii*) Mass transport for pH-responsive nanocarriers;
- (*iii*) Mass transport for the free, bound, and internalized drug; and.
- (*iv*) Fraction of killed cells parameter which is calculated based on intracellular drug concentration.

**Figure 5:**
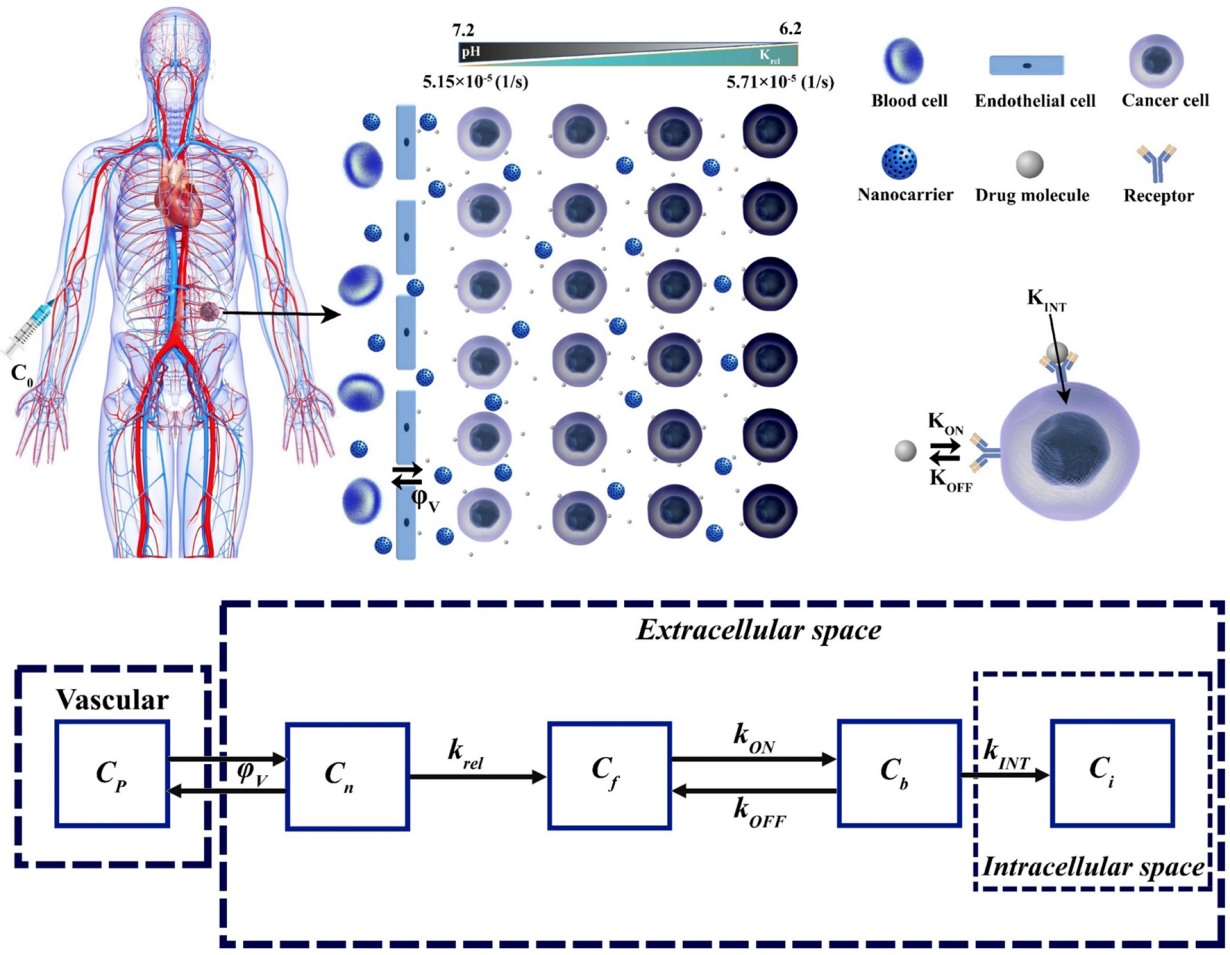
A schematic of drug transport in the vascular, interstitium, and intracellular spaces along with its compartmental representation. (${C}_{0}$: Initial concentration , ${C}_{p}$: Vascular concentration , ${C}_{n}$: Nanocarrier concentration, ${C}_{f}$: Free drug concentration, ${C}_{b}$: Bound drug concentration, ${C}_{i}$: Intracellular concentration, ${k}_{rel}$: Constant of the drug release , ${k}_{ON}$: Rate of association of drug with receptors of the cell-surface, ${k}_{OFF}$: Rate of disassociation of drug with receptors of the cell-surface, ${k}_{INT}$: Constant of cellular uptake,${\varphi }_{V}$: Rate of drug transport per unit volume through the microvessels into the interstitium).

The dynamic process in drug delivery includes nanocarrier exchange between blood microvessels and interstitium, drug release in interstitium, and association/disassociation of drug with receptors of the cell-surface at the rate constants of ${K}_{ON}$ and ${K}_{\mathrm{OFF}}$, respectively. Besides, the last stage is the influx of drugs from extracellular space to cancer cells. In the present study, a multi-compartmental model has been used for mathematical modeling, which enables quantifying the biochemical and physiological phenomena. In compartment models, the distribution of drugs in each compartment is dependent on both spatial and temporal variations. The general block diagram of the current study considering nanocarriers and chemotherapeutic drugs is shown in Fig. 5.

**Figure 6:**
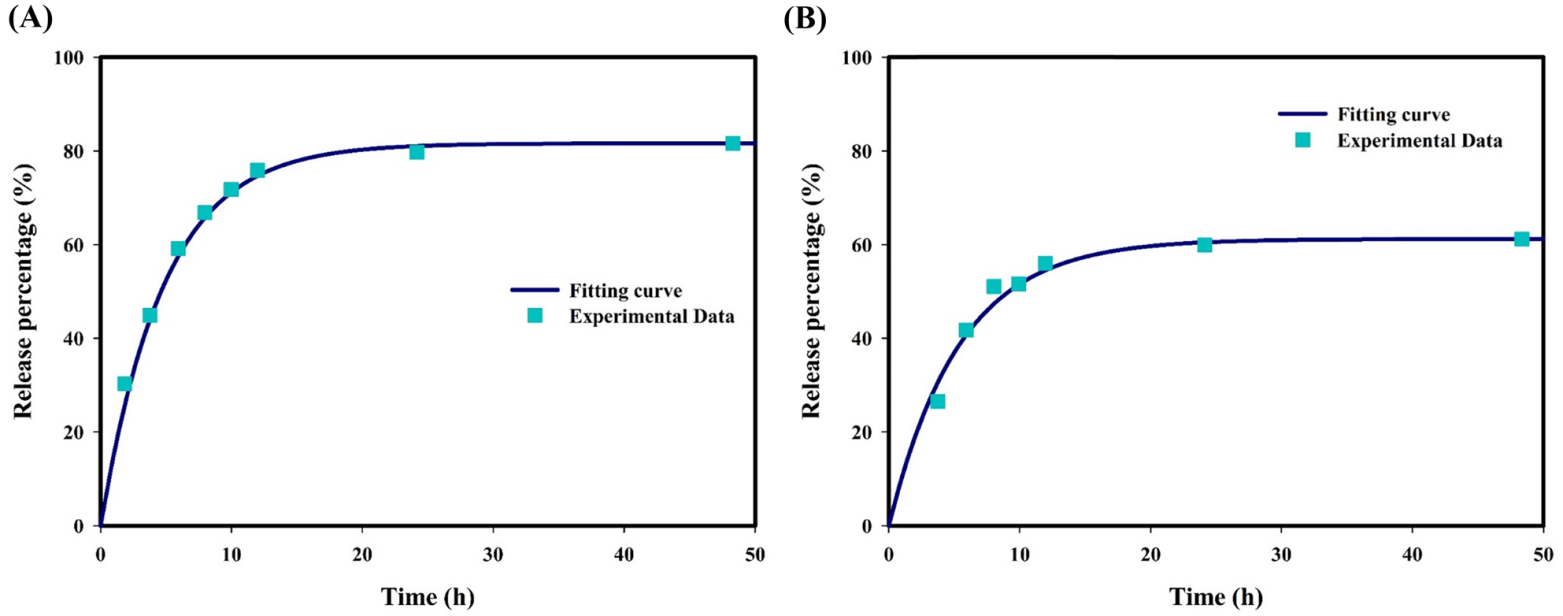
Drug release at (**A**) pH = 6.2; and (**B**) pH = 7.2. Experimental data extracted from the literature^38^.

In the present study, the binding of the drug to the protein in the interstitium was neglected according to the high binding affinity $\left({K}_{ON}/{K}_{\mathrm{OFF}}\right)$ of the drug to cell-surface receptors. Additionally, at neutral pH, the drug release rate is considered zero; so, there is no free drug in the circulatory system and microvascular network. The relation between release percentage and time of exposure is found to follow the first-order kinetics represented as^9,37^:

1 $$\%R\left(t\right)={R}_{c}(1-\mathrm{exp}(-{k}_{rel}t))$$

where ${R}_{c}$ and $\%R(t)$ are the total percentage of drug released at a given pH level and the percentage of drug released at exposure time $t$, respectively. This equation is used to fit experimental data obtained in the pH range of 6 to 7.2^38^ and curve fitting for drug release at pH of 6.2 and 7.2, as shown in Fig. 6.

From the best curve fitting results, release rates at different pH values in the range of 6.2 to 7.2 are summarized in Table 1. Linear interpolation is performed to obtain the release rate at pH value between the listed pH points.

**Table 1:** Release rates at various pH levels.

pH	6.2	6.4	6.6	6.8	7	7.2
${k}_{rel} [1/s]$	$5.71\times {10}^{-5}$	$5.59\times {10}^{-5}$	$5.48\times {10}^{-5}$	$5.37\times {10}^{-5}$	$5.26\times {10}^{-5}$	$5.15\times {10}^{-5}$

According to the physics of biological tissues, tissue is considered as a porous environment; so, the Darcy law is employed to solve the fluid flow in tissue. Accordingly, the fluid flow in interstitium can be defined as^39^:

2 $${v}_{i}=-\kappa \nabla {P}_{i}$$

where ${v}_{i}$ and ${P}_{i}$ denoted IFV and IFP, respectively.$\kappa$ is defined as $k/\mu$ that denoted the coefficient of interstitial hydraulic conductivity. Additionally, $k$ and $\mu$ illustrates permeability of tissue and the dynamic viscosity of fluid, respectively. Assuming the existence of source and sink terms in tissue due to the blood microvessels and lymphatic drainage system, the continuity equation is corrected as^31^:

3 $$\nabla \cdot {v}_{i}={\varnothing }_{V}-{\varnothing }_{L}$$

in which ${\varnothing }_{V}$ is the fluid flow rate from the microvascular network to the interstitial space and ${\varnothing }_{L}$ is the fluid flow rate from interstitial space to lymphatic microvessels, defined as^31^:

4 $${\varnothing }_{V}=\frac{{L}_{P}S}{V}({P}_{v}-{P}_{i}-{\sigma }_{s}\left({\pi }_{B}-{\pi }_{i}\right))$$

5 $${\varnothing }_{L}={L}_{PL}{(\frac{S}{V})}_{L}({P}_{i}-{P}_{L})$$

In which *S/V* is the surface area per unit volume of capillaries that its value varies for tumor tissue depending on the location. ${L}_{PL}{(\frac{S}{V})}_{L}$ is the lymph filtration coefficient and ${P}_{L}$ is lymph hydrostatic pressure. Lymphatic drainage system is just considered in normal tissue based on the literature^31,39,40^.

To reach tumor site, the nanocarriers and its therapeutic agents can be existed in various forms as: nanocarriers in the interstitial space (${C}_{n}$), free drugs in the interstitial space (${C}_{f}$), bound drugs (${C}_{b}$), and internalized drugs to cells (${C}_{i}$)^41,42^. Systemically administered drug-loaded nanocarriers are transported to tumor sites through the circulatory system, undergo transvascular extravasation followed by distribution in the interstitium, release their cargo in interstitium, and then binding to cancer cells and cellular internalization are happened. For transport of nanocarriers containing chemotherapeutic agents, the system of equations is adjusted thus^43^:

6 $$\frac{\partial {C}_{n}}{\partial t}=-{v}_{i}\nabla {C}_{n}+{D}_{n}{\nabla }^{2}{C}_{n}-{K}_{rel}{C}_{n}+({\varphi }_{V}-{\varphi }_{L})$$

7 $$\frac{\partial {C}_{f}}{\partial t}=\alpha {K}_{rel}{C}_{n}-{v}_{i}\nabla {C}_{f}+{D}_{f}{\nabla }^{2}{C}_{f}-\frac{1}{\varphi }{K}_{ON}{C}_{rec}{C}_{f}+{K}_{OFF}{C}_{b}$$

8 $$\frac{\partial {C}_{b}}{\partial t}=\frac{1}{\varphi }{K}_{ON}{C}_{rec}{C}_{f}-{K}_{OFF}{C}_{b}-{K}_{INT}{C}_{b}$$

9 $$\frac{\partial {C}_{i}}{\partial t}={K}_{INT}{C}_{b}$$

where $${K}_{rel}$$, $${D}_{n}$$, $${D}_{f}$$, $$ {C}_{rec}$$, and $${K}_{INT}$$ are respectively the constant of the drug release rate from the carrier, the carrier diffusion coefficient, the free drug diffusion coefficient, concentration of cell-surface receptors, constant of cellular uptake rate. $$\alpha $$ is the number of chemotherapy agents contained in the nanocarrier. In the present study, it is assumed that the number of drugs molecule loaded for nanocarriers in different sizes (20–100 nm) is equal, so the $$\alpha $$ parameter for each is considered 20.

In Eq. (5), ${\varphi }_{V}$ is the rate of drug transport per unit volume through the microvessels into the interstitium and ${\varphi }_{L}$ is the rate of drug transport per unit volume from the interstitium into the lymph system. ${\varphi }_{V}$ is described, due to pore model, as^36,42^:

10 $${\varphi }_{V}={\varnothing }_{V}\left(1-{\sigma }_{f}\right){C}_{P}+\frac{{P}_{n}S}{V}({C}_{P}-{C}_{n})\frac{Pe}{{e}^{Pe}-1}$$

11 $$Pe=\frac{{\varnothing }_{V}(1-{\sigma }_{f})}{{P}_{n}S/V}$$

where ${\sigma }_{f}$,${C}_{P}$, and ${P}_{n}$ are the filtration reflection coefficient, the injected drug concentration, and is the vascular permeability coefficient, respectively. $Pe$ is the Peclet number, illustrating the convection transport rate to the diffusion transport rate. ${\varphi }_{L}$ has been assumed to be as follow^36,42^:

12 $${\varphi }_{L}={\varnothing }_{L}{C}_{n}$$

A bolus injection of drug-loaded pH-responsive nanocarriers, representing the vascular concentration, is defined as^44^:

13 $${C}_{P}={C}_{0}\mathrm{exp}(-t/{K}_{d})$$

where ${C}_{0}$ and ${K}_{d}$ are the initial concentration and blood circulation decay constant, respectively.

### Fraction of killed cells

The anticancer effects is determined by the fraction of killed cell parameter using an empirical equation for drug doxorubicin^45^. The fraction of killed cells, which depends on *C*_*i*_, is obtained as^46^:

14 $${S}_{f}=1-\mathrm{exp}(-\omega \cdot {C}_{i})$$

In which ω is a fitting parameter determined for doxorubicin based on the experiments^47^.

Table 2 demonstrates the interstitial transport parameters for both tumor and healthy tissues. Moreover, Table 3 illustrates both the nanocarriers and doxorubicin transport parameters for tumor and healthy tissues.

### Solution strategy and boundary conditions

In this study, a solid tumor with a radius of 1 cm is considered within a normal tissue that is 3 times the tumor tissue. The computational domain is considered as a 2D-axisymmetric geometry. The boundary conditions of this study are mentioned in Table 4. For solving this problem, there are two different steps including steady-state and time-dependent. By solving Darcy's law in the steady-state step the IFP and IFV are obtained. Afterward, time-dependent solute transport equations are solved. The coupled nonlinear set of the above-mentioned governing equations and also the boundary conditions are assessed through the finite element method. The simulation is performed using the commercial finite element software COMSOL Multiphysics 5.5 (COMSOL, Inc., Burlington, MA, USA). A segregated approach is applied to solve the equations with the time-step of 0.1 s and relative tolerance of 0.001. Time of analysis for nanocarrier-mediated drug delivery are considered as 240 h.

**Table 2:** Parameters of interstitial fluid transport.

Parameter	Unit	Description	Value	Ref
${P}_{v}$	$Pa$	Vascular fluid pressure	$2080$ (Normal)	^44^
			$$2080$$ (Tumor)	
${\pi }_{v}$	$Pa$	Oncotic pressure of microvessels	$2666$ (Normal)	^31^
			$$2666$$ (Tumor)	
${\pi }_{i}$	$Pa$	Oncotic pressure of interstitium	$1333$ (Normal)	^31^
			$$2000$$ (Tumor)	
${\sigma }_{s}$	-	Average coefficient of osmotic reflection	$0.91$ (Normal)	^31^
			$$0.82$$ (Tumor)	
${L}_{p}$	$m/Pa\cdot s$	Hydraulic conductivity of the microvessel wall	$2.1\times {10}^{-11}$ (Normal)	^31^
			$$2.7\times {10}^{-12}$$ (Tumor)	
${L}_{PL}{(\frac{S}{V})}_{L}$	$1/(Pa\cdot s)$	Lymphatic filtration coefficient	$4.17\times {10}^{-7}$ (Normal)	^36,44^
			0 (Tumor)	
$\kappa$	${cm}^{2}/(mmHg\cdot s)$	Hydraulic conductivity of interstitium	$8.53\times {10}^{-9}$ (Normal)	^36,44^
			$$4.13\times {10}^{-8}$$ (Tumor)	
${P}_{L}$	$Pa$	Hydrostatic pressure of lymph microvessels	0	^36,44^

**Table 3:** Parameters for nanocarriers and doxorubicin transport.

Parameter	Unit	Description		Value	Ref
${P}_{n}$	$m/s$	Vascular permeability of nanocarriers	20 nm	$4.6\times {10}^{-10}$ (Tumor)	^48,49^
			50 nm	$7.38\times {10}^{-11}$ (Tumor)	
			100 nm	$5.44\times {10}^{-12}$ (Tumor)	
${D}_{n}$	${m}^{2}/s$	Diffusion coefficient of nanocarriers	20 nm	$7\times {10}^{-12}$ (Tumor)	^48,49^
			50 nm	$4.4\times {10}^{-12}$ (Tumor)	
			100 nm	$2.2\times {10}^{-12}$ (Tumor)	
${\sigma }_{f}$		Reflection coefficient	20 nm	0.043 (Tumor)	^48,49^
				$$1$$ (Normal)	
			50 nm	0.227 (Tumor)	
				$$1$$ (Normal)	
			100 nm	0.634 (Tumor)	
				$$1$$ (Normal)	
${K}_{d}$	$min$	Blood circulation decay constant	20 nm	1320	^48,49^
			50 nm	1096	
			100 nm	600	
${D}_{f}$	${m}^{2}/s$	Diffusion coefficient of free drug	$1.58\times {10}^{-10}$ (Normal)		^48,49^
			$$3.4\times {10}^{-10}$$ (Tumor)		
K_ON_	${m}^{3}/(mole\cdot s)$	Binding rate constant	300		^48,49^
K_OFF_	$1/s$	Unbinding rate constant	$8\times {10}^{-3}$		^48,49^
K_INT_	$1/s$	Internalization rate constant	$5\times {10}^{-5}$		^48,49^
*φ*	-	Tumor volume fraction available to drugs	$0.05$		^48,49^
C_rec_	$M$	Concentration of cell-surface receptors	$1\times {10}^{-5}$		^48,49^
*ω*	${m}^{3}/mole$	Survival constant of cancer cells	$0.6603$		^48,49^

**Table 4:** Boundary conditions used in this study.

Region	Boundary conditions for	
	Fluid flow	Concentration
Tumor center	$\nabla {P}_{i}=0$	$D\nabla C+{v}_{i}C=0$
Inner boundary	$-{k}_{t}\nabla {P}_{i}{\Omega }^{-}=-{k}_{n}\nabla {P}_{i}{\Omega }^{+}$	$\left({D}_{t}\nabla C+{v}_{i}C\right){\Omega }^{-}=\left({D}_{n}\nabla C+{v}_{i}C\right){\Omega }^{+}$
	${P}_{i}{\Omega }^{-}={P}_{i}{\Omega }^{+}$	$C{\Omega }^{-}=C{\Omega }^{+}$
Outer boundary	${P}_{i}$= Constant	$-n\cdot \nabla \mathrm{C}=0$

${\Omega }^{-}$ and ${\Omega }^{+}$ demonstrates the tumor and healthy tissue at the boundary, respectively.

## Data Availability

All data used for this study are available from the author upon request.

## References

[1] Grimes DR, Jansen M, Macauley RJ, Scott JG, Basanta D (2020). Evidence for hypoxia increasing the tempo of evolution in glioblastoma. Br. J. Cancer.

[2] DaiPhung C, Tran TH, Nguyen HT, Jeong J-H, Yong CS, Kim JO (2020). Current developments in nanotechnology for improved cancer treatment, focusing on tumor hypoxia. J. Controlled Release.

[3] Sørensen BS, Horsman MR (2020). Tumor hypoxia: impact on radiation therapy and molecular pathways. Front. Oncol..

[4] Folkman J (1986). How is blood vessel growth regulated in normal and neoplastic tissue?. GHA Clowes Mem. Award Lecture Cancer Res.

[5] Bergers G, Benjamin LE (2003). Tumorigenesis and the angiogenic switch. Nat. Rev. Cancer.

[6] Stylianopoulos T, Jain RK (2013). Combining two strategies to improve perfusion and drug delivery in solid tumors. Proc. Natl. Acad. Sci..

[7] Stylianopoulos T, Munn LL, Jain RK (2018). Reengineering the physical microenvironment of tumors to improve drug delivery and efficacy: from mathematical modeling to bench to bedside. Trends Cancer.

[8] Huang S, Michalek JE, Reardon DA, Wen PY, Floyd JR, Fox PT, Clarke GD, Jerabek PA, Schmainda KM, Muzi M (2021). Assessment of tumor hypoxia and perfusion in recurrent glioblastoma following bevacizumab failure using MRI and 18 F-FMISO PET. Sci. Rep..

[9] F.M. Kashkooli, M. Soltani, M. Souri, Controlled anti-cancer drug release through advanced nano-drug delivery systems: Static and dynamic targeting strategies. J. Controlled Release (2020). 10.1016/j.jconrel.2020.08.012 32800878

[10] Parks SK, Chiche J, Pouysségur J (2013). Disrupting proton dynamics and energy metabolism for cancer therapy. Nat. Rev. Cancer.

[11] P. Vaupel, Oxygen supply to malignant tumors, Tumor blood circulation: angiogenesis, vascular morphology and blood flow of experimental and human tumors, CRC Press2020, pp. 143–168.

[12] Kumari R, Sunil D, Ningthoujam RS (2020). Hypoxia-responsive nanoparticle based drug delivery systems in cancer therapy: an up-to-date review. J. Control. Release.

[13] Denny WA (2000). The role of hypoxia-activated prodrugs in cancer therapy. Lancet Oncol..

[14] Wilson WR, Hay MP (2011). Targeting hypoxia in cancer therapy. Nat. Rev. Cancer.

[15] Brown JM, Wilson WR (2004). Exploiting tumour hypoxia in cancer treatment. Nat. Rev. Cancer.

[16] Semenza GL (2010). Defining the role of hypoxia-inducible factor 1 in cancer biology and therapeutics. Oncogene.

[17] Carmeliet P, Jain RK (2000). Angiogenesis in cancer and other diseases. Nature.

[18] Burgdorf S, Porubsky S, Marx A, Popovic ZV (2020). Cancer acidity and hypertonicity contribute to dysfunction of tumor-associated dendritic cells: potential impact on antigen cross-presentation machinery. Cancers.

[19] V. Huber, C. Camisaschi, A. Berzi, S. Ferro, L. Lugini, T. Triulzi, A. Tuccitto, E. Tagliabue, C. Castelli, L. Rivoltini, Cancer acidity: An ultimate frontier of tumor immune escape and a novel target of immunomodulation. Sem Cancer Biol Elsevier, 2017, pp. 74–89. 10.1016/j.semcancer.2017.03.001 28267587

[20] Corbet C, Feron O (2017). Tumour acidosis: from the passenger to the driver's seat. Nat. Rev. Cancer.

[21] Damaghi M, Wojtkowiak JW, Gillies RJ (2013). pH sensing and regulation in cancer. Front. Physiol..

[22] F.M. Kashkooli, M. Soltani, M. Souri, C. Meaney, M. Kohandel, Nexus between in silico and in vivo models to enhance clinical translation of nanomedicine, Nano Today, 36 (2021) 101057.

[23] Sarin H (2010). Physiologic upper limits of pore size of different blood capillary types and another perspective on the dual pore theory of microvascular permeability. J. Angiogenes. Res..

[24] Liu J, Huang Y, Kumar A, Tan A, Jin S, Mozhi A, Liang X-J (2014). pH-sensitive nano-systems for drug delivery in cancer therapy. Biotechnol. Adv..

[25] M.J. Mitchell, M.M. Billingsley, R.M. Haley, M.E. Wechsler, N.A. Peppas, R. Langer, Engineering precision nanoparticles for drug delivery. Nat. Rev. Drug Discov. (2020) 1–24. 10.1038/s41573-020-0090-8 PMC7717100 33277608

[26] Sun T, Zhang YS, Pang B, Hyun DC, Yang M, Xia Y (2014). Engineered nanoparticles for drug delivery in cancer therapy. Angew. Chem. Int. Ed..

[27] Engin K, Leeper D, Cater J, Thistlethwaite A, Tupchong L, McFarlane J (1995). Extracellular pH distribution in human tumours. Int. J. Hyperth..

[28] Stubbs M, McSheehy PM, Griffiths JR, Bashford CL (2000). Causes and consequences of tumour acidity and implications for treatment. Mol. Med. Today.

[29] Stylianopoulos T, Jain RK (2015). Design considerations for nanotherapeutics in oncology, nanomedicine: Nanotechnology. Biol. Med..

[30] Chauhan VP, Stylianopoulos T, Martin JD, Popović Z, Chen O, Kamoun WS, Bawendi MG, Fukumura D, Jain RK (2012). Normalization of tumour blood vessels improves the delivery of nanomedicines in a size-dependent manner. Nat. Nanotechnol..

[31] Soltani M, Chen P (2011). Numerical modeling of fluid flow in solid tumors. PloS One.

[32] M. Soltani, Numerical modeling of drug delivery to solid tumor microvasculature, (2013). 10.1016/j.mvr.2015.02.007 25724978

[33] M. Al-Zu’bi, A. Mohan, Modelling of combination therapy using implantable anticancer drug delivery with thermal ablation in solid tumor. Sci. Rep. 10 (2020) 1–16. 10.1038/s41598-020-76123-0 PMC7653950 33168846

[34] Boucher Y, Jain RK (1992). Microvascular pressure is the principal driving force for interstitial hypertension in solid tumors: implications for vascular collapse. Can. Res..

[35] Raghunathan S, Evans D, Sparks JL (2010). Poroviscoelastic modeling of liver biomechanical response in unconfined compression. Ann. Biomed. Eng..

[36] Kashkooli FM, Soltani M, Rezaeian M, Meaney C, Hamedi M-H, Kohandel M (2020). Effect of vascular normalization on drug delivery to different stages of tumor progression: In-silico analysis. J. Drug Delivery Sci. Technol..

[37] Zhan W, Gedroyc W, Xu XY (2019). Towards a multiphysics modelling framework for thermosensitive liposomal drug delivery to solid tumour combined with focused ultrasound hyperthermia. Biophysics Reports.

[38] Lee ES, Na K, Bae YH (2003). Polymeric micelle for tumor pH and folate-mediated targeting. J. Control. Release.

[39] Baxter LT, Jain RK (1989). Transport of fluid and macromolecules in tumors. I. Role of interstitial pressure and convection. Microvascular Res..

[40] Kashkooli FM, Soltani M, Hamedi M-H (2020). Drug delivery to solid tumors with heterogeneous microvascular networks: Novel insights from image-based numerical modeling. Eur. J. Pharmaceut. Sci..

[41] Kashkooli FM, Soltani M, Rezaeian M, Taatizadeh E, Hamedi M-H (2019). Image-based spatio-temporal model of drug delivery in a heterogeneous vasculature of a solid tumor—computational approach. Microvasc. Res..

[42] Soltani M, Sefidgar M, Bazmara H, Casey ME, Subramaniam RM, Wahl RL, Rahmim A (2017). Spatiotemporal distribution modeling of PET tracer uptake in solid tumors. Ann. Nucl. Med..

[43] Mpekris F, Angeli S, Pirentis AP, Stylianopoulos T (2015). Stress-mediated progression of solid tumors: effect of mechanical stress on tissue oxygenation, cancer cell proliferation, and drug delivery. Biomech. Model. Mechanobiol..

[44] F.M. Kashkooli, M. Soltani, M.M. Momeni, A. Rahmim, Enhanced drug delivery to solid tumors via drug-loaded nanocarriers: An image-based computational framework. Front. Oncol. (2021). 10.3389/fonc.2021.655781 PMC8264267 34249692

[45] Kerr DJ, Kerr AM, Freshney RI, Kaye SB (1986). Comparative intracellular uptake of adriamycin and 4'-deoxydoxorubicin by nonsmall cell lung tumor cells in culture and its relationship to cell survival. Biochem. Pharmacol..

[46] Mpekris F, Baish JW, Stylianopoulos T, Jain RK (2017). Role of vascular normalization in benefit from metronomic chemotherapy. Proc. Natl. Acad. Sci..

[47] Kerr D, Kerr A, Freshney R, Kaye S (1986). Delivery of molecular and cellular medicine to solid tumors. Biochem Pharmacol.

[48] Stylianopoulos T, Economides E-A, Baish JW, Fukumura D, Jain RK (2015). Towards optimal design of cancer nanomedicines: multi-stage nanoparticles for the treatment of solid tumors. Ann. Biomed. Eng..

[49] Chauhan VP, Stylianopoulos T, Martin JD, Popović Z, Chen O, Kamoun WS, Bawendi MG, Fukumura D, Jain RK (2020). Normalization of tumour blood vessels improves the delivery of nanomedicines in a size-dependent manner.

